# ΔNp63 Controls a TLR3-Mediated Mechanism That Abundantly Provides Thymic Stromal Lymphopoietin in Atopic Dermatitis

**DOI:** 10.1371/journal.pone.0105498

**Published:** 2014-08-29

**Authors:** Terufumi Kubo, Ryuta Kamekura, Ayako Kumagai, Koji Kawata, Keiji Yamashita, Yukari Mitsuhashi, Takashi Kojima, Kotaro Sugimoto, Akihiro Yoneta, Yasuyuki Sumikawa, Toshiharu Yamashita, Noriyuki Sato, Tetsuo Himi, Shingo Ichimiya

**Affiliations:** 1 Department of Pathology, Sapporo Medical University School of Medicine, Sapporo, Japan; 2 Department of Otolaryngology, Sapporo Medical University School of Medicine, Sapporo, Japan; 3 Department of Human Immunology, Research Institute for Frontier Medicine, Sapporo Medical University School of Medicine, Sapporo, Japan; 4 Department of Dermatology, Sapporo Medical University School of Medicine, Sapporo, Japan; 5 Department of Cell Science, Research Institute for Frontier Medicine, Sapporo Medical University School of Medicine, Sapporo, Japan; University Hospital Hamburg-Eppendorf, Germany

## Abstract

In the skin lesions of atopic dermatitis (AD), keratinocytes release large quantities of thymic stromal lymphopoietin (TSLP), causing unfavorable inflammation along with skin damage. Nevertheless, how TSLP influences keratinocytes themselves is still unknown. In this study, we showed that ΔNp63, a p53-homologue, predominantly expressed in keratinocytes regulated the receptor complex of TSLP, which determines susceptibility to self-derived TSLP. Expression of TSLP receptors in skin tissues and keratinocytes was assessed by immunohistochemistry and quantitative RT-PCR, and *in vitro* studies were also performed to examine the functional relevance of ΔNp63 in the expression of TSLP receptors and the constituting autocrine and/or paracrine pathway of TSLP under the condition of stimuli to innate receptors sensing cell damage. The results showed that normal keratinocytes in the upper epidermis preferentially expressed TSLP receptors and conversely lacked ΔNp63, which has an inhibitory effect on the expression of TSLP receptors. Interestingly, the epidermis of AD lesions was found to abundantly contain keratinocytes with low or undetectable levels of ΔNp63 (ΔNp63^lo/-^). Moreover, in the absence of ΔNp63, keratinocytes readily presented TSLP and other cytokines by stimuli through Toll-like receptor 3 (TLR3). Together with the evidence that extrinsic TSLP itself augments TSLP production by keratinocytes without ΔNp63, the results indicate that ΔNp63^lo/-^ keratinocytes generate TSLP through a putative autocrine and/or paracrine pathway upon TLR3 stimulation within AD lesions, since moieties of damaged cells and pathogens stimulate TLR3.

## Introduction

The prevalence of allergic disorders has been significantly increasing in many countries in the past few decades. The fact that over 10% of children and 6% of adults suffer from atopic dermatitis (AD) has become a worldwide health issue [1]. Due to intense intractable pruritus, patients with AD primarily experience sleep disturbances, which often lead to a significant decline of daily activities and growth retardation. Later in life, children suffering from AD will frequently be affected by other allergic disorders such as allergic asthma or rhinitis, following a process known as the ‘atopic march’ [2], [3]. Since the mechanism of AD has not been fully elucidated, an understanding of the process underlying AD should help to formulate appropriate therapies not only for improvement in quality of life but also for prevention of AD-related allergic disorders.

Extensive investigations have revealed a salient abnormality of AD skin lesions in which an interleukin (IL)-7-like cytokine, thymic stromal lymphopoietin (TSLP), produced by keratinocytes provokes a hyperreactive immune state [4], [5], [6]. In AD lesions, TSLP activates myeloid dendritic cells and then promotes the differentiation of naïve CD4^+^ T cells into T helper type 2 (Th2) cells secreting IL-4, IL-5 and IL-13, in a manner similar to that in allergic asthma. TSLP is also able to stimulate eosinophils, macrophages, invariant natural killer T cells and innate lymphoid cells, eventually resulting in chronic inflammation. Recent genome-wide association studies on AD patients have revealed somatic mutations of genes regulating keratinocytes such as those for filaggrin, a natural skin moisturizer, and claudin-1, a representative barrier protein [7], [8], [9]. Functional loss of filaggrin disrupts the epidermal skin barrier and consequently causes keratinocytes to produce TSLP [10]. However, the filaggrin gene mutation is observed in only about 30% of AD patients and is also detected in patients with other inflammatory skin diseases such as ichthyosis vulgaris. Thus, the dysfunction of filaggrin is not sufficient to explain the mechanism underlying TSLP overproduction by keratinocytes.

Transcription regulator p63 (also called keratinocyte transcription factor, KET), a p53 homologue, plays a primary role in the construction of fully functional epidermal layers [11], [12]. p63 exerts different functions by virtue of its various isoforms. ΔNp63, which lacks an N-terminal transactivation domain, is widely expressed in epidermal keratinocytes to form the skin barrier in concert with filaggrin and involcurin, and it controls the expression of claudin-1 as well as proinflammatory cytokines [13], [14]. However, whether ΔNp63 has a role in an inflammatory environment such as AD skin lesions remains elusive.

Here we report the abundance of unique keratinocytes that expressed ΔNp63 at low or undetectable levels (collectively named ΔNp63^lo/-^ keratinocytes) within AD lesions and an interesting role of ΔNp63 in the production of TSLP. Studies of normal epidermal tissues and keratinocyte-derived cells have demonstrated that loss of ΔNp63 increased the level of TSLP receptors, which are composed of a heterodimer of TSLP-binding chain (TSLPR) and interleukin-7 receptor α (IL-7Rα) chain. Interestingly, stimulation of TLR3 by poly I:C downregulated ΔNp63 and upregulated TSLP receptors in keratinocytes, which concomitantly produced TSLP. This process related to TLR3 was found to be dependent on nuclear factor κB (NF-κB). Given that damaged cell-derived self-RNA can stimulate TLR3 signaling, these findings imply that ΔNp63^lo/-^ keratinocytes in AD lesions may cause chronic inflammation through a vicious cycle of their own TSLP-TSLPR axis loop induced by RNA moieties derived from surrounding damaged keratinocytes [15], [16].

## Materials and Methods

### Tissues

Skin tissues were obtained from patients with AD or systemic lupus erythematosus (SLE) and patients presenting without pathological findings who underwent skin biopsies at Sapporo Medical University Hospital. The diagnosis of AD was established by both dermatologists and pathologists. All tissues were obtained with written informed consent according to the guidelines of the Declaration of Helsinki and with approval of the Institutional Review Board (IRB) of Sapporo Medical University Hospital under permit number 25–136 titled investigation of immunologic abnormality in inflammatory skin diseases.

### Cell cultures

Human primary keratinocytes were purchased from DS Pharma Biomedical (Tokyo, Japan) and cultured in a serum-free medium for human keratinocytes (DS Pharma Biomedical). Human HaCaT epidermal cells (RIKEN, Tsukuba, Japan) were maintained in DMEM (Sigma-Aldrich, St Louis, MO) supplemented with 100 U/ml penicillin G, 100 µg/ml streptomycin and 5% fetal bovine serum. For stimulation of cells, culture media were supplemented with the following reagents: 10 µg/ml zymozan (InvivoGen, San Diego, CA), 5 to 25 µg/ml polyinosine-polycytidylic acid (poly I:C; InvivoGen), 100 ng/ml lipopolysaccharide (LPS; InvivoGen), 5 µM Type B CpG oligonucleotide (ODN2006; InvivoGen), 10 ng/ml TNFα (R&D Systems, Minneapolis, ME), or 1 to 10 ng/ml TSLP (R&D Systems). All cells were cultured at 37°C in a humidified atmosphere in 5% CO_2_.

Cells were treated with poly I:C at 5 µg/ml (for primary keratinocytes) or 10 µg/ml (for HaCaT keratinocytes) and TSLP (final concentration 1 or 10 ng/ml) simultaneously. The culture media was changed into the media with poly I:C and TSLP at 16 hr after p63 siRNA transfection. For detection of TSLP protein by ELISA, we removed the media and washed cells with PBS twice at 24 hr after poly I:C and TSLP stimulation. And then the cells were cultured in the media containing only poly I:C at the same concentration for 48 hr. For detection of GM-CSF or IL-6 protein by ELISA, culture supernatant were collected at 48 hr after poly I:C and TSLP treatment.

### Antibodies

The primary antibodies used were a mouse monoclonal antibody (mAb) to detect all isoforms of p63 (4A4; Dako, Glostrup, Denmark), a mouse anti-TSLPR mAb (147036; R&D Systems), a mouse anti-β-actin mAb (AC-15; Sigma-Aldrich), a rabbit polyclonal antibody (pAb) to detect ΔNp63 (p40; Merck KGaA, Darmstadt, Germany), a rabbit anti-IL-7Rα pAb (Rockland, Gilbertsville, PA), a rabbit anti-TSLP pAb (Abcam, Cambridge, MA), a sheep anti-TSLP pAb (R&D Systems), a rabbit anti-cytokeratin pAb (Biogenesis, Poole, England), a rabbit anti-NF-κB p65 mAb (D14E12; Cell Signaling, Beverly, MA) and a rabbit anti-phospho NF-κB p65 (Ser536) mAb (93H1; Cell Signaling). Alexa 488 (green)- and Alexa 594 (red)-conjugated anti-mouse and anti-rabbit IgGs were purchased from Invitrogen (Carlsbad, CA). Peroxidase-conjugated goat anti-mouse and anti-rabbit IgGs were obtained from KPL (Gaithersburg, MD).

### Quantitative RT-PCR analyses

Total RNA was extracted and reverse-transcribed into cDNA as described previously [17]. Quantitative RT-PCR was performed as described in the manufacturer's protocol for Assays-on-Demand Gene Expression products (Life Technologies, Carlsbad, CA). The amount of glyceraldehyde 3-phosphate dehydrogenase (GAPDH) mRNA in each sample was used to standardize the quantities of p63 mRNA (Hs00978344), p73 mRNA (Hs01056230), both long and short transcript variants of TSLP mRNA (Hs00263639), TSLPR mRNA (Hs00845692), IL-7Rα mRNA (Hs00902334), IL-6 mRNA (Hs00174131) and GM-CSF mRNA (Hs00929873). To calculate the relative mRNA expression of triplicate specimens, the ΔΔCT method was employed according to the manufacturer's instructions.

### siRNA transfection

A cocktail of three siRNAs for human p63 (siTrio full set) was purchased from B-Bridge International (Sunnyvale, CA) and transfected at 100 nmol/L. The sense sequences of the siRNAs for human p63 were 5′-CAGAAGAUGGUGCGACAAATT-3′, 5′-GUGAAUUCAACGAGGGACATT-3′, and 5′-GCAAAAAAGAGUUGGGUGUTT-3′. Negative control siRNA was obtained from Invitrogen. Transfections were carried out using Lipofectamine RNAi max (Invitrogen) in Opti-MEM (GIBCO, Carlsbad, CA) according to the manufacturer's instructions. Briefly, 7.5 µl of Lipofectamine RNAi max and siRNA for p63 were suspended into 500 µl Opti-MEM each. After mixing them together, Opti-MEM containing Lipofectamine RNAi max and the siRNA was incubated at room temperature for 15 min and then added into 2 ml culture medium. The culture medium was changed at 16 hr after transfection.

### Immunostaining analysis

Formalin-fixed and paraffin-embedded and frozen tissue sections were used for immunostaining analysis as described previously [17], [18]. Specimens were examined with a laser scanning confocal microscope (FV300, Olympus, Tokyo, Japan) or a fluorescence microscope (IX71, Olympus). To investigate tissue sections after immunostaining with anti-p63 antibodies, nuclear levels of ΔNp63 were classified into three categories by staining intensity, as indicated by ΔNp63^hi^, ΔNp63^lo^ and ΔNp63^−^. Three pathologists independently examined a specimen and determined the ratio of ΔNp63^lo/-^ cells per total cells in at least 5 high-power fields. Final scores of a specimen were defined by averages.

### Immunoblot analysis

Procedures of cell lysis and immunoblotting were as described previously [17]. The levels of intensity of signals detected in immunoblots were quantified using NIH Image-J software. The intensity levels were normalized to the corresponding levels of β-actin, and their relative levels were shown in histograms.

### Enzyme-linked immunosorbent assay (ELISA)

Cells were cultured at a density of 1×10^5^ cells/well in a 12-well plate under the condition. Concentrations of TSLP, GM-CSF and IL-6 in culture supernatants were analyzed in triplicate with specific ELISA kits (R&D Systems) as described in the manufacturer's protocol. Detection limit of TSLP, GM-CSF and IL-6 are 9.87 pg/ml, 3 pg/ml and 10 pg/ml, respectively.

### TSLP blocking experiment

Cells were cultured at a density of 5×10^5^ cells/well in a 12-well plate before stimulation. The cells were treated with 2 µg/ml anti-TSLP pAb (R&D Systems) and/or 5 µg/ml poly I:C simultaneously 24 hr after plating them and incubated for 24 hr. They were harvested for total RNA isolation. Sheep IgG was used as a negative control.

### TRIF inhibition experiment

HaCaT keratinocytes were plated at a density of 1×10^6^ cells/well in a 6-well plate. After 24 hr, medium was changed and the cells were treated with 5 µM TRIF inhibitory peptide or 5 µM control peptide (Invivogen) for 16 hr. The cells were irradiated with 60 mJ/cm^2^ of UVB. The cells were harvested and total RNA was isolated at 24 hr after irradiation.

### Statistical analysis

Statistical significance was examined using the unpaired two-tailed Student's *t* test or one-way ANOVA followed by Tukey's multiple-comparison test. Graph bars in the figures present means ± SD.

## Results

### ΔNp63 influences the levels of a TSLP receptor complex

**Figure 1:**
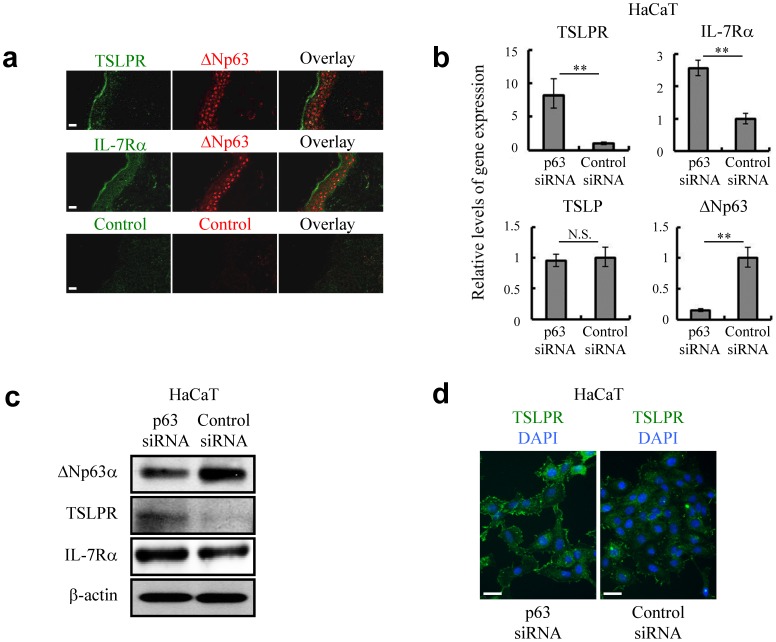
Expression of TSLPR and IL-7Rα in epidermal keratinocytes is controlled by ΔNp63. (**a**) Immunofluorescence labeling confocal microscopy to localize TSLPR and IL-7Rα in the area of epidermis where ΔNp63 was not detected. Bar  = 20 µm. (**b**, **c**) Quantitative RT-PCR (**b**) and immunoblot analysis (**c**) against ΔNp63, TSLPR, IL-7Rα and TSLP in control siRNA (siControl) and p63-specific siRNA (sip63)-transfected HaCaT keratinocytes. β-actin was used as a loading control. The cells were harvested for assays at 72 hr after siRNA transfection. Student's *t* test. **P<0.01 and N.S., not significant. (**d**) Immunofluorescence labeling fluorescence microscopy to localize TSLPR in siControl and sip63 HaCaT keratinocytes. The cells were fixed at 72 hr after siRNA introduction. DAPI, 4′,6-diamidino-2-phenylindole. Bar  = 50 µm. Data are representative of at least three independent experiments.

Normal epidermal tissues predominantly express ΔNp63 among the several isoforms of transcription factor p63 (Figure S1a) [19], [20]. Along with terminal differentiation of keratinocytes from basal cells to superficial cells in the normal epidermis, the expression level of nuclear ΔNp63 gradually decreases and disappears in the superficial layers to characterize physiological cellular function [21], [22]. Interestingly, keratinocytes residing in the superficial layers with low or undetectable levels of ΔNp63 abundantly expressed TSLPR and IL-7Rα, which form a heterodimeric complex for TSLP (Figure 1a). This suggests that keratinocytes comprising the superficial layers are likely to be more sensitive to surrounding TSLP than are keratinocytes in the basal layers, and the mechanism of expression of TSLP receptors would be related to ΔNp63. Further *in vitro* studies using HaCaT and primary keratinocytes with p63-specific siRNA revealed that downregulation of ΔNp63 could increase the expression of TSLPR and IL-7Rα, even though keratinocytes presented certain levels of TSLPR and IL-7Rα (Figures 1b, 1c and Figure S2). Indeed, loss of ΔNp63 prompted upregulation of TSLPR on the cell boundaries (Figure 1d). Treatment with a conditioned medium derived from ΔNp63-knockdown cells did not solely alter the expression levels of these receptors (data not shown). Therefore, soluble factors would not mediate between ΔNp63 and TSLP receptors. Taken together, we concluded that the level of ΔNp63 affects the expression of a TSLP receptor complex. Since the level of ΔNp63 itself did not have the capacity to affect levels of TSLP in keratinocytes (Figure 1b), this raised the question of whether there is a factor(s) inducing the production of TSLP by keratinocytes.

### TLR3 stimulation represses expression of ΔNp63 and promotes production of TSLP

**Figure 2:**
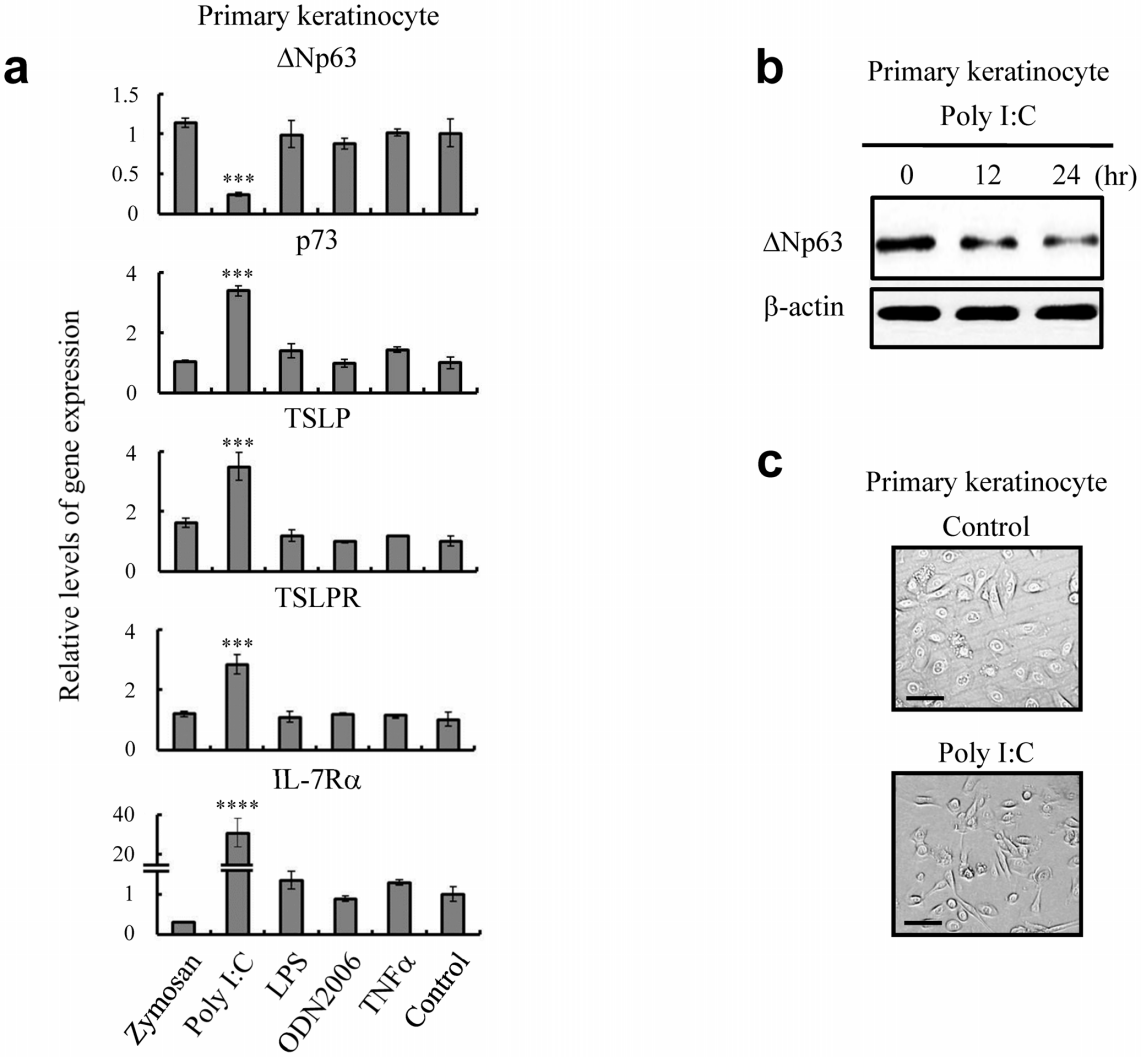
TLR3 stimulation alters the expression levels of AD-related molecules in primary keratinocytes. (**a**) Quantitative RT-PCR demonstrating the levels of ΔNp63, p73, TSLP, TSLPR and IL-7Rα mRNAs at 24 hr after treatment with 25 µg/ml poly I:C. One-way ANOVA followed by Tukey's multiple-comparison test. ***P<0.005 and ****P<0.001 versus control. (**b**) Immunoblot analysis showing the level of ΔNp63 at different time course after treatment with 25 µg/ml poly I:C. (**c**) Phase-contrast microscopy demonstrating the morphological changes of primary keratinocytes at 24 hr after 25 µg/ml poly I:C treatment. Bar  = 50 µm. Data are representative of three independent experiments.

Many investigations have suggested that epidermal cells that are responsible for the first line of defense employ innate sensors such as Toll-like receptor (TLR) family members for the emergence of efficient immunity [23], [24]. To examine the effect of TLRs on the production of TSLP, we stimulated keratinocytes with various specific ligands for TLRs. As shown in Figures 2a and 2b, treatment with a TLR3 ligand of poly I:C at a subtoxic dose (25 µg/ml) exclusively increased the TSLP mRNA and decreased the level of ΔNp63 mRNA. More interestingly, poly I:C stimulation was solely sufficient to decrease the expression of ΔNp63 and induce TSLP receptors as well as p73, a p53-like molecule, suggesting that the expression of ΔNp63, TSLP, and p73 is under the control of a TLR3 signaling pathway (Figure 2a). TLR3 signaling is known to be induced by ultraviolet (UV) irradiation, under which keratinocytes also showed a phenotype change for expression of ΔNp63 and TSLP receptors similar to that obtained by stimulation with poly I:C (Figure S3a) [16]. To investigate the role of TLR3 signaling in the regulation of ΔNp63, we pretreated keratinocytes with an inhibitor of TIR-domain-containing adaptor-inducing interferon-β (TRIF), an adaptor responding to the activation of TLR3. The reduction of ΔNp63 by UV irradiation was clearly interfered with the TRIF inhibitor (Figure S3b). These results imply that TLR3 signaling naturally regulates ΔNp63-mediated cellular response of keratinocytes, which might be associated with TSLP production. Moreover, TLR3 stimulation seemed to abrogate intercellular adhesive activities, similar to the manner of detachment of ΔNp63-deficient epidermal cells at the skin surface (Figure 2c and Figure S1b). Collectively, the results indicate that a TLR3-mediated pathway stimulated by poly I:C and other possible ligands such as double-stranded (ds) RNA might have the capacity to decrease expression levels of ΔNp63 in keratinocytes and simultaneously promote their TSLP production.

### TLR3 stimulation enhances a TSLP-dependent inflammatory circuit

**Figure 3:**
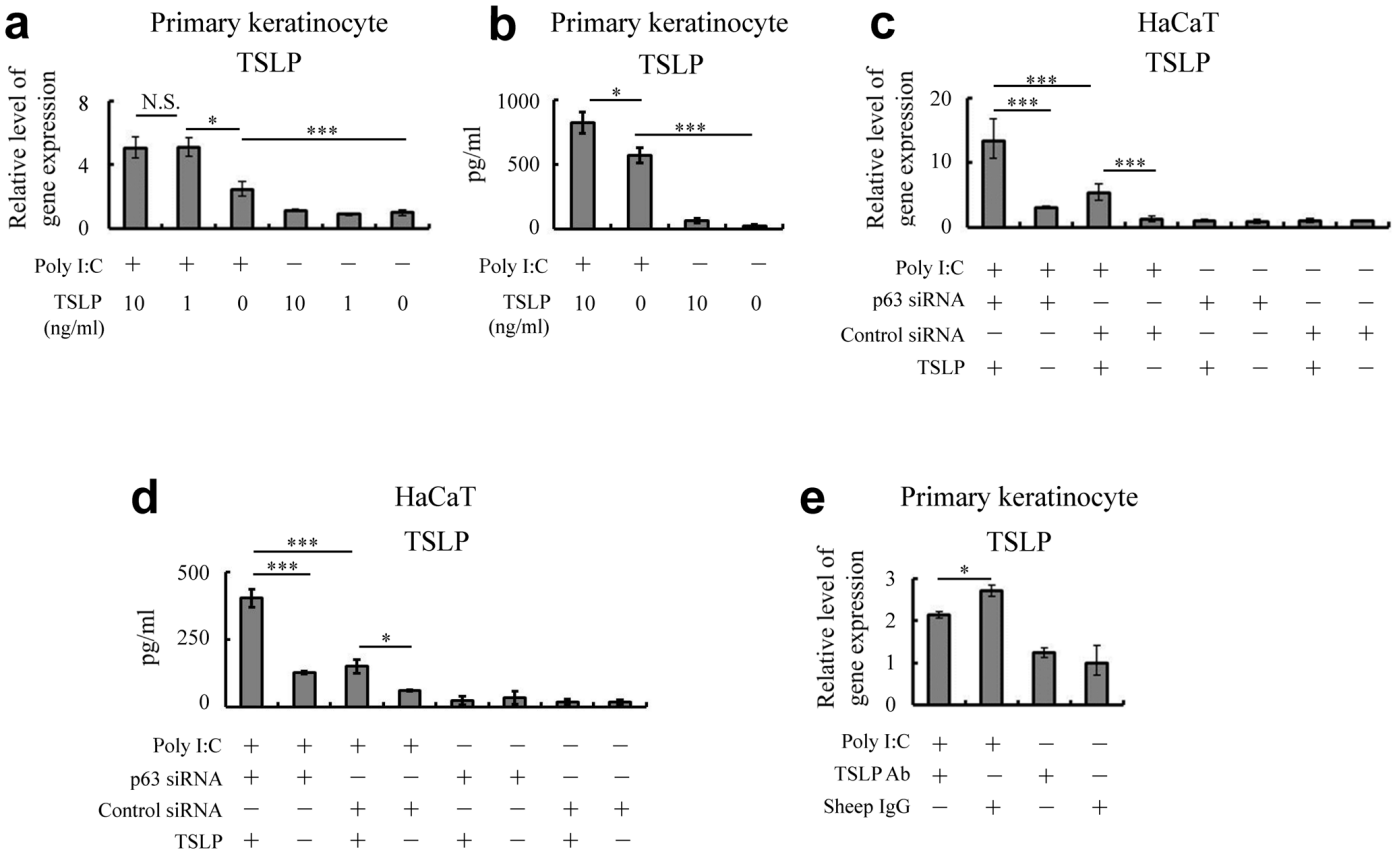
TSLP enhances inflammatory responses in ΔNp63-deficient keratinocytes. (**a, b**) Quantitative RT-PCR (**a**) and ELISA (**b**) demonstrate the level of endogenous TSLP at 24 hr (**a**) or 72 hr (**b**) after treatment with exogenous TSLP (1 or 10 ng/ml) under the condition of TLR3 stimulation (5 µg/ml poly I:C) in primary keratinocytes. (**c, d**) Quantitative RT-PCR (**c**) and ELISA (**d**) show the level of endogenous TSLP at 24 hr (**c**) or 72 hr (**d**) after stimulation with 10 µg/ml poly I:C and 10 ng/ml TSLP in siControl and sip63 HaCaT keratinocytes. (**e**) Quantitative RT-PCR confirming whether neutralization of TSLP inhibits the generation of TSLP under the condition of TLR3 stimulation (5 µg/ml poly I:C) in primary keratinocytes. One-way ANOVA followed by Tukey's multiple-comparison test. *P<0.05, ***P<0.005 and N.S., not significant. Data are representative of at least three independent experiments.

**Figure 4:**
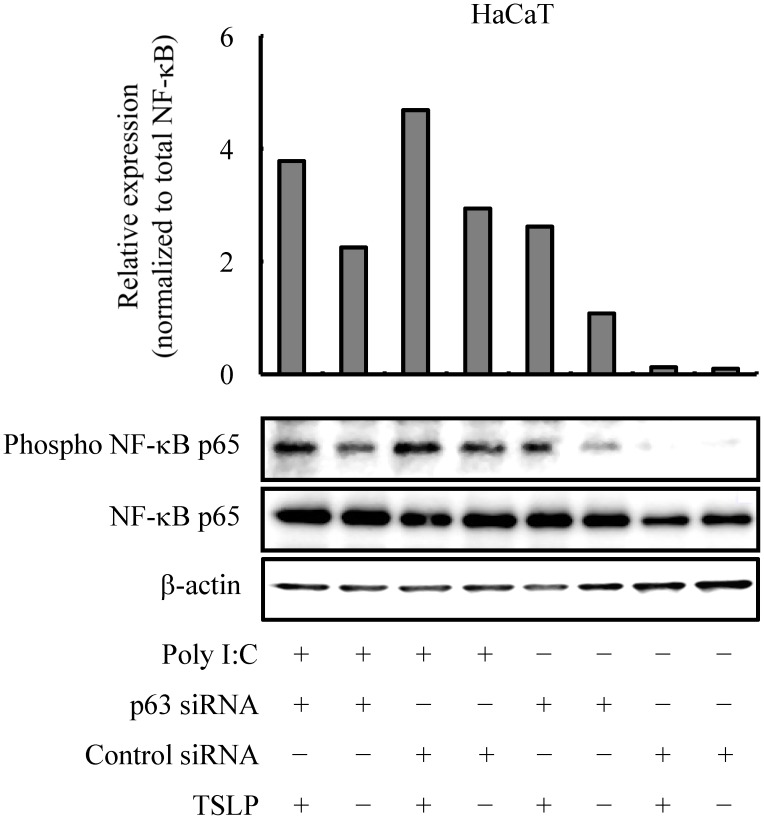
TSLP induces signal transduction in ΔNp63-deficient HaCaT keratinocytes. Immunoblot analysis demonstrating phosphorylation of the p65 subunit of NF-κB at 24 hr after treatment with 10 µg/ml poly I:C and exogenous TSLP (10 ng/ml). The histogram shows the relative phospho- NF-κB expression normalized to NF-κB as determined by densitometric analysis. β-actin was used as a loading control. Data are representative of three independent experiments.

Next, we examined the effects of exogenous TSLP on keratinocytes stimulated with TLR3. Results demonstrated that exogenous TSLP helped the keratinocytes under the condition of TLR3 stimulation to produce intrinsic TSLP (Figures 3a–d), as well as inflammatory cytokines such as granulocyte-macrophage colony-stimulating factor (GM-CSF) and IL-6 (Figure S4a–c). When keratinocytes were pretreated with p63-specific siRNA, cellular responses to exogenous TSLP under the condition of TLR3 stimulation enhanced the production of these cytokines (Figures 3c, 3d and Figure S4b and S4c). More interestingly, treatment of the culture supernatant with TSLP-specific antibodies decreased the levels of these cytokines in keratinocytes under the same condition of TLR3 stimulation (Figure 3e and Figure S4d), implying that keratinocytes in this condition possessed a TSLP-related autocrine and/or paracrine mechanism. As assessed by immunoblot analysis, the p65 subunit of NF-κB was phosphorylated after treatment with the TLR3 ligand of poly I:C and exogenous TSLP (Figure 4). This suggested that ΔNp63-deficient keratinocytes are larger producers of TSLP and inflammatory cytokines in response to TLR3 ligands and exogenous TSLP as well, at least in part, through the NF-κB pathway.

### AD skin lesions preferentially contain ΔNp63^lo/-^ keratinocytes

**Figure 5:**
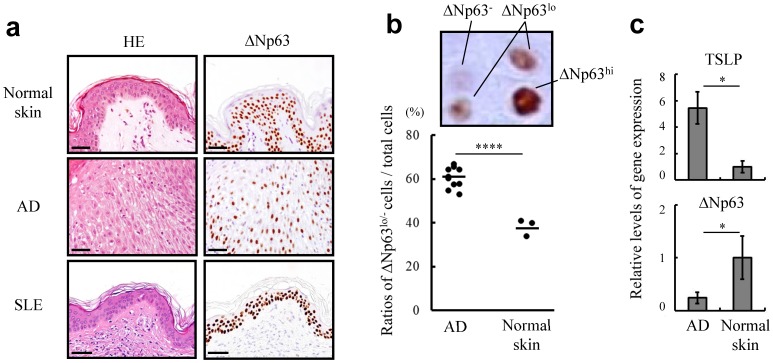
Unique expression profiles of ΔNp63 in AD epidermal lesions. (**a**) Immunohistochemical staining of ΔNp63 in normal skin and skin lesions of AD and SLE. Consecutive sections of each condition were stained with hematoxylin and eosin (HE). Bar  = 50 µm. (**b**) Level of ΔNp63 was classified into three categories depending on staining intensity, as indicated by ΔNp63^hi^, ΔNp63^lo^ and ΔNp63^−^. The dot plot represents ratios of ΔNp63^lo/-^ keratinocytes in normal skin (n = 3) and AD lesions (n = 10). (**c**) Quantitative RT-PCR demonstrating the levels of TSLP and ΔNp63 mRNAs in normal skin and AD lesions. Student's *t* test. *P<0.05 and ****P<0.001. Data are representative of at least three independent experiments.

We further investigated the expression profiles of ΔNp63 in skin lesions of patients with AD by immunohistochemistry. It was of great interest that the AD skin lesions were composed of keratinocytes with variable expression of ΔNp63, i.e., a mixture of cells with ΔNp63 at high, low or undetectable levels (ΔNp63^hi^, ΔNp63^lo^ or ΔNp63^−^, respectively) in the epidermal areas. In contrast, the epidermal areas of normal skin tissues as well as skin lesions of patients with SLE were composed of keratinocytes steadily expressing ΔNp63 at high levels (Figure 5a) [14]. Indeed, the ratios of ΔNp63^lo^ and ΔNp63^−^ (ΔNp63^lo/-^) keratinocytes to total keratinocytes of the AD skin lesions were much higher than those in normal epidermal tissues (Figure 5b, Figures S5 and S6). Accordingly, analysis of the epidermal tissue of an AD lesion by quantitative RT-PCR showed that the expression level of ΔNp63 was reduced in comparison to that in normal skin (Figure 5c). Collectively, we conclude that AD lesions likely harbor ΔNp63^lo/-^ keratinocytes within epidermal areas, though the reason why ΔNp63^lo/-^ keratinocytes reside in AD lesions is still unclear. The results together with results from *in vitro* studies suggesting a functional relation of ΔNp63 and TLR3 stimulation indicate that ΔNp63^lo/-^ keratinocytes might provide a potent autocrine and/or paracrine loop to generate TSLP and promote chronic inflammation.

## Discussion

**Figure 6:**
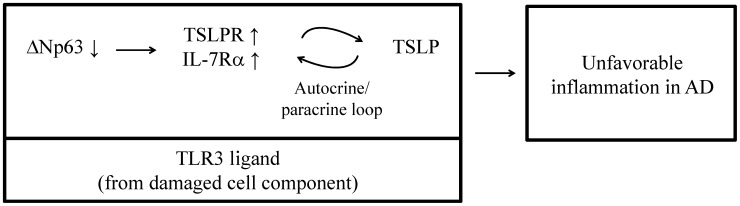
Schematic diagram of findings in this study. AD lesions possess many ΔNp63^lo/-^ keratinocytes, which might be considered as a major source of TSLP. A potential TLR3 ligand such as damaged-cell-derived dsRNA suppresses the expression of ΔNp63, which eventually further upregulates TSLPR and IL-7Rα and augments the susceptibility to TSLP in ΔNp63^lo/-^ keratinocytes. Since TSLP itself can help ΔNp63^lo/-^ keratinocytes to produce endogenous TSLP under the condition of TLR3 stimulation, an autocrine and/or paracrine loop of TSLP are generated in ΔNp63^lo/-^ keratinocytes, leading to undesired inflammation of AD lesions.

Accumulating evidence supports the idea that p53-homologous transcription factors such as p63 and p73 regulate epithelial cell integrity, as indicated by findings that the balance of production and degradation of p53 homologues determines cell fate in response to a variety of noxious stimuli such as ultraviolet irradiation and chemotherapeutics [11], [25], [26]. Of note, epidermal keratinocytes actively defend themselves against various external stimuli as well as internal substances derived from keratinizing apoptotic cells [27]. Thus, p53 homologues can be considered to have a role in the control of immunological responses of epidermal tissues. We previously reported the functional significance of p53 homologues in human tissues such as tonsils and the medullary region of the thymus, in which epithelial cells express TSLP [18], [28], [29]. Therefore, these findings imply that the production of TSLP is associated with the function of p53 homologues in AD lesions. To the best of our knowledge, this study is the first study showing that TSLP receptors are physiologically presented by superficial keratinocytes of normal skin tissues and their expression is associated with ΔNp63. However, it is still unclear how ΔNp63 influences transcription of the genes encoding TSLPR and IL-7Rα. We also demonstrated that the epidermal lesions of AD preferentially contain a large amount of ΔNp63^lo/-^ keratinocytes. Within the AD lesions, ΔNp63^lo/-^ keratinocytes might sense TSLP through TSLP receptors and, in response to TSLP, could have the capacity to further produce TSLP and other inflammatory cytokines. Our results reveal a putative autocrine and/or paracrine pathway of TSLP production in AD skin lesions that would prolong local inflammation and eventually contribute to the establishment of the inflammatory lesions of AD. Our results further demonstrated that TLR3 stimulation of such cells robustly led to the downregulation of ΔNp63 and upregulation of TSLP receptors. Considering these findings collectively, we propose a TLR3-related vicious circle underlying the overproduction of TSLP in AD skin lesions as summarized in Figure 6. The present study could not fully reveal the pathological significance of p73 in AD lesions. We have to elucidate them in the future study.

Recent studies have demonstrated that, for minimizing tissue damage due to self-injuries, innate receptors have a cardinal role in the response to moieties featuring damage-associated molecular patterns. During this process, TLR3 is in charge of sensing extracellular dsRNA derived from damaged cells [15], [16]. In lesions of AD, keratinocytes reside around damaged cells, where dsRNA might downregulate ΔNp63. Previous studies have shown that cellular damage or treatment with TLR3 ligands induces the production of TSLP in keratinocytes [6], [30]. Given that a transgenic mouse model of keratinocyte-specific overproduction of TSLP is very similar to the chronic eczematous lesions of AD [31], aberrant production of TSLP followed by cell damage and liberation of dsRNA might underlie the clinical state of AD. Taken together with our observation that downregulation of ΔNp63 through TLR3 signaling enhanced susceptibility to self- or surrounding keratinocytes-derived TSLP by means of TSLP receptor upregulation, it appears that cell-damage-related dsRNA may play a fundamental role in the pathogenesis of AD. In this context, loss of function mutation in the filaggrin gene breaks the epithelial barrier and causes further cell damage, which could be brought about by repeated scratching behavior or infection, leading to deterioration of AD through activation of such a TLR3-dependent mechanism [32], [33]. We could not confirm the upregulation of TSLP/GM-CSF/IL-6 at transcript and protein levels in ΔNp63-deficient keratinocytes treated with TSLP and without poly I:C stimulation (Figures 3a–d, Figures S4a–c). Intracellular signals mediated by TLR3 may underlie its mechanism, suggesting that keratinocytes which is not sensing self-damage via TLR3 cannot react to TSLP in order to avoid spreading of further unnecessary inflammation. While UV radiation of the skin to eliminate inflammatory cells is an effective modality for treatment of AD, sunburn often causes deterioration of the skin condition of patients with AD [34], [35], [36]. Therefore, the intensity and duration of UV exposure are critical for the treatment of AD. Excess exposure to UV radiation induces damage of keratinocytes and releases cellular TLR3 ligands, probably leading to activation of the autocrine and/or paracrine loop of TSLP production.

Conventional strategies for treatment of AD chiefly rely on local administration of a moisturizing agent and/or immunosuppressants as pallative care [37]. The former sustains and reinforces the barrier function of epidermal tissues, while the latter suppresses the activity of effector cells to stop inflammation. According to the results of this study, a modality to inhibit TLR3 signaling or upregulate ΔNp63 of keratinocytes would be effective for treatment of AD, because ΔNp63 works as a key molecule translating cellular insults into immune responses as well as barrier functions.

In summary, AD lesions are characterized by an abundance of ΔNp63^lo/-^ keratinocytes, which are assumed to play a role in the overproduction of TSLP through a TLR3-related autocrine and/or paracrine loop. The expression level of ΔNp63 determines the level of TSLP receptors and the ability to recognize surrounding damaged cells via TLR3. A new modality to inhibit this loop could lead to an effective treatment of AD.

## Supporting Information

Figure S1 **Keratinocytes abundantly express ΔNp63.** (**a**) Immunoblot analysis showing the expressions of ΔNp63 and TAp63 in epidermal keratinocytes. Epidermal keratinocytes predominantly express ΔNp63, but not TAp63. Other isoforms of p63 in the keratinocytes were undetectable by immunoblot analysis. β-actin was used as a loading control. (**b**) Phase-contrast microscopy demonstrating the morphological features of intercellular adhesive properties of ΔNp63-deficient HaCaT keratinocytes dramatically changed a cohesive pattern into a scattered pattern. Bar  = 100 µm.
(TIFF)

Figure S2 **Expression of TSLPR and IL-7Rα is regulated by ΔNp63 in primary keratinocytes.** Quantitative RT-PCR demonstrating the levels of ΔNp63, TSLPR and IL-7Rα mRNAs in control siRNA (siControl) and p63-specific siRNA (sip63)-transfected primary keratinocytes. Downregulation of ΔNp63 by specific siRNA increased the levels of TSLPR and IL-7Rα mRNAs. Student's *t* test. *P<0.05 and ***P<0.005 versus control. Data are representative of three independent experiments.
(TIFF)

Figure S3 **Effects of ultraviolet B (UVB) irradiation on HaCaT keratinocytes.** (**a**) Quatitative RT-PCR showing the levels of ΔNp63, TSLPR and IL-7Rα mRNAs in UVB-exposed HaCaT keratinocytes. UVB exposure simply reduced the level of ΔNp63 and concomitantly upregulated the levels of TSLPR and IL-7Rα mRNAs. (**b**) Quantitative RT-PCR confirming whether inhibition of Toll/interleukin-1 receptor domain-containing adaptor protein inducing interferon β (TRIF) rescues downregulation of ΔNp63 mRNA by UVB exposure in HaCaT keratinocytes. TRIF inhibitor rescued ΔNp63 downregulation by UVB exposure. One-way ANOVA followed by Tukey's multiple-comparison test.*P<0.05, **P<0.01 and ***P<0.005 versus control. Data are representative of at least three independent experiments.
(TIFF)

Figure S4 **Expression of inflammatory cytokines in ΔNp63-deficient keratinocytes is enhanced by TSLP stimulation.** (**a**) Quantitative RT-PCR demonstrating the levels of granulocyte colony-stimulating factor (GM-CSF) and IL-6 mRNAs at 24 hr after treatment with exogenous TSLP (1 or 10 ng/ml) under the condition of TLR3 stimulation (5 µg/ml poly I:C) in primary keratinocytes. Treatment with exogenous TSLP increased the expressions of these cytokines under the condition of TLR3 stimulation. (**b**) Quantitative RT-PCR showing the levels of GM-CSF and IL-6 mRNAs at 24 hr after stimulation with 10 µg/ml poly I:C and 10 ng/ml TSLP in siControl and sip63 HaCaT keratinocytes. Stimulation with TLR3 and TSLP enhanced the expressions of these cytokines in ΔNp63-deficient HaCaT keratinocytes. (**c**) ELISA demonstrating GM-CSF and IL-6 release at 48 hr after treatment with 5 µg/ml poly I:C and 10 ng/ml TSLP in culture supernatant from siControl and sip63 primary keratinocytes. Down regulation of ΔNp63 and/or exogenous TSLP increase GM-CSF and IL-6 release under the condition of TLR3 stimulation. (**d**) Quantitative RT-PCR confirming whether neutralization of TSLP inhibits the generation of GM-CSF and IL-6 under the condition of TLR3 stimulation (5 µg/ml poly I:C) in primary keratinocytes. Neutralization of TSLP inhibits the expressions of these cytokines under the condition of TLR3 stimulation. One-way ANOVA followed by Tukey's multiple-comparison test. *P<0.05, ***P<0.005 and N.S., not significant. Data are representative of at least three independent experiments.
(TIFF)

Figure S5 **Representative figures of ΔNp63-immunostaining of AD skin lesions.** Paraffin-embedded tissue sections of 10 cases of AD lesions (AD1 to AD10) were examined for the distribution of ΔNp63. AD10 is depicted in Figure 5a and the cases of AD1 to AD9 are shown here. Bar  = 20 µm.
(TIFF)

Figure S6 **Immunofluorescence labeling of ΔNp63 and TSLP in AD and normal skin.** TSLP was overexpressed in the skin from a patient with AD compared to the level in normal skin. On the other hand, keratinocytes in the skin from a patient with AD expressed a lower level of ΔNp63 than that in normal keratinocytes. Bar  = 20 µm.
(TIFF)
