# Supplementary figures and images for: ΔNp63 Controls a TLR3-Mediated Mechanism That Abundantly Provides Thymic Stromal Lymphopoietin in Atopic Dermatitis
Source: PLoS One. 2014 Aug 29;9(8):e105498. doi: 10.1371/journal.pone.0105498 (PMC4149429; doi:10.1371/journal.pone.0105498)

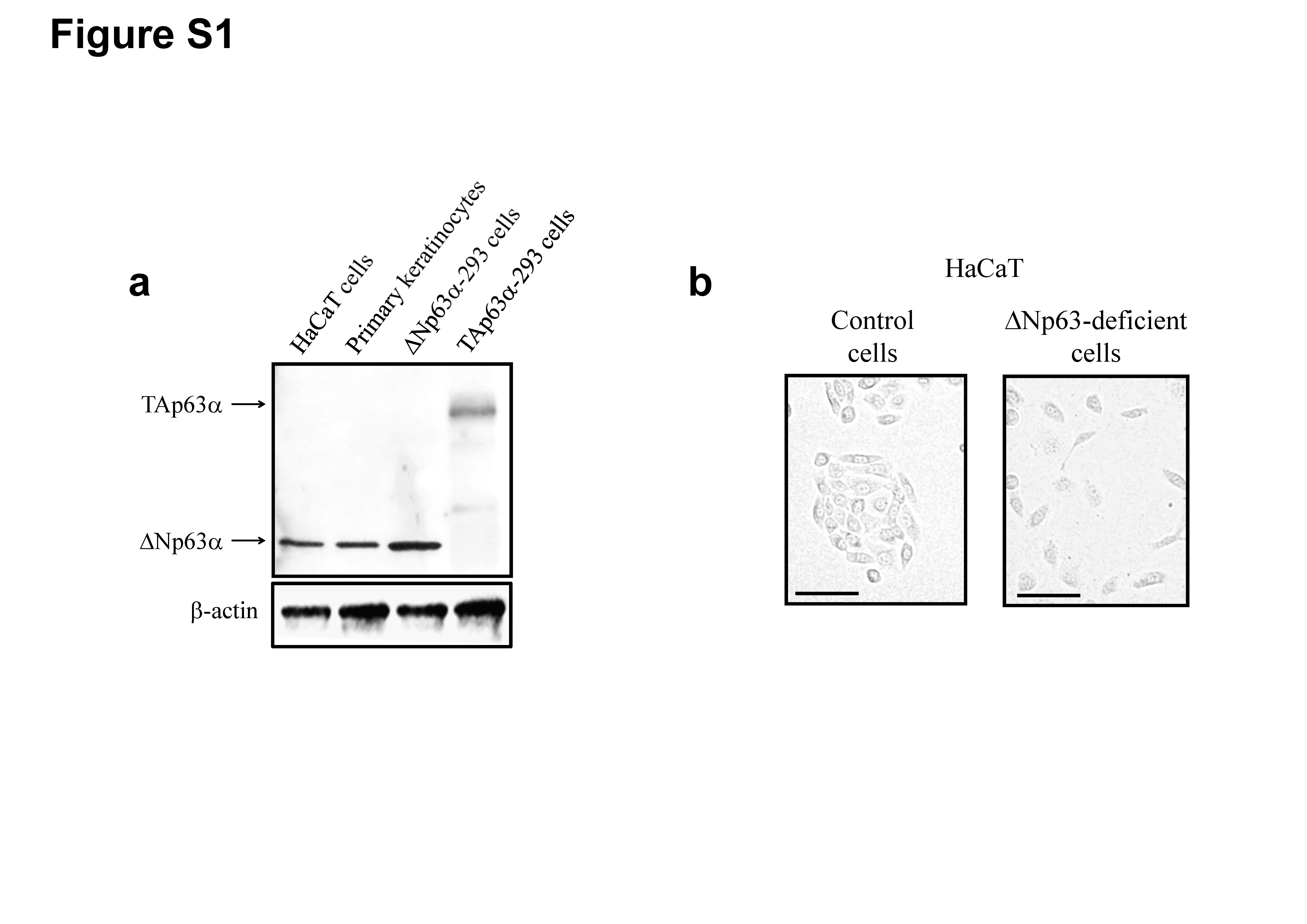

Supplement: Figure S1 — Keratinocytes abundantly express ΔNp63. (a) Immunoblot analysis showing the expressions of ΔNp63 and TAp63 in epidermal keratinocytes. Epidermal keratinocytes predominantly express ΔNp63, but not TAp63. Other isoforms of p63 in the keratinocytes were undetectable by immunoblot analysis. β-actin was used as a loading control. (b) Phase-contrast microscopy demonstrating the morphological features of intercellular adhesive properties of ΔNp63-deficient HaCaT keratinocytes dramatically changed a cohesive pattern into a scattered pattern. Bar = 100 µm. (TIFF) [file pone.0105498.s001.tiff]

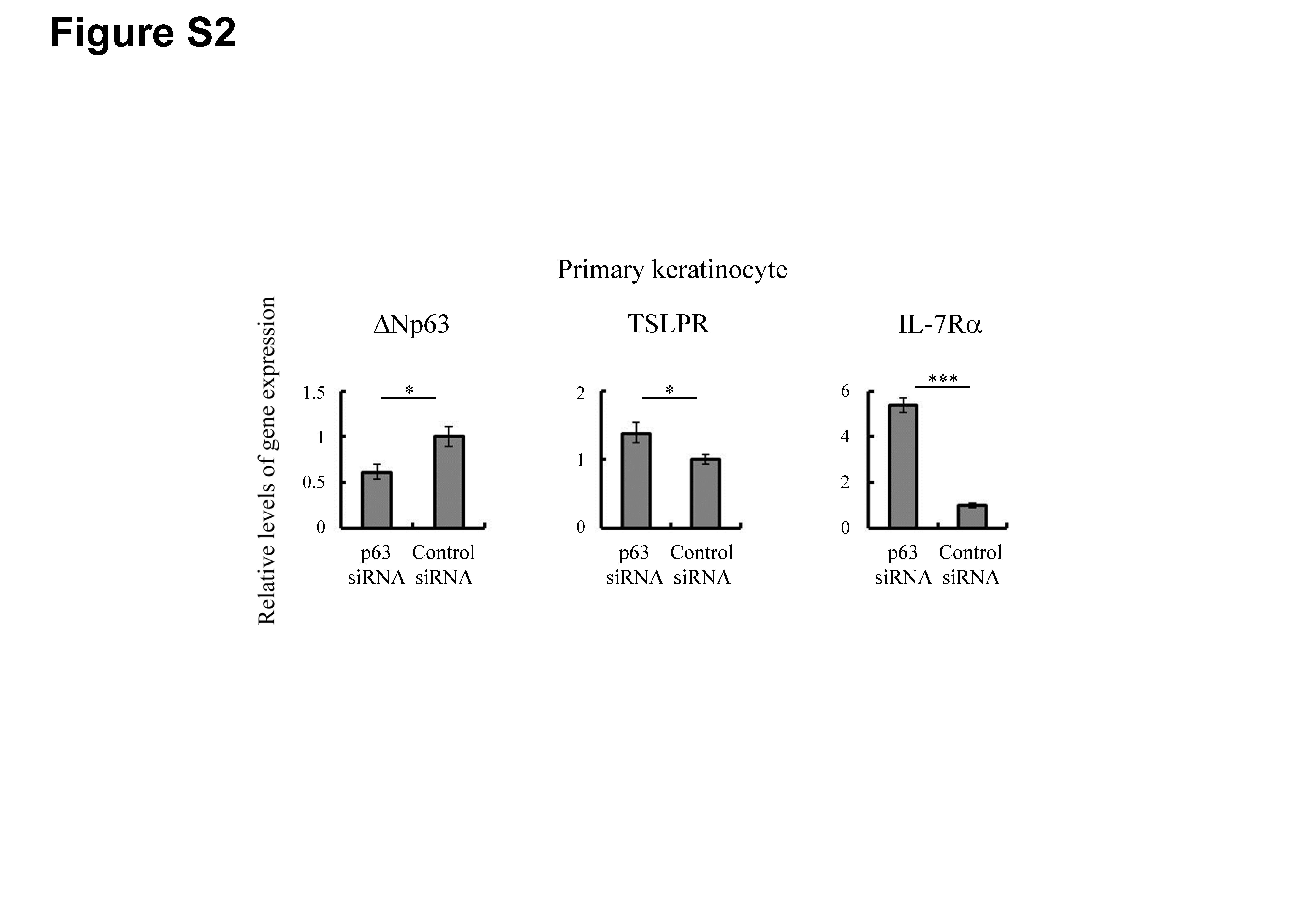

Supplement: Figure S2 — Expression of TSLPR and IL-7Rα is regulated by ΔNp63 in primary keratinocytes. Quantitative RT-PCR demonstrating the levels of ΔNp63, TSLPR and IL-7Rα mRNAs in control siRNA (siControl) and p63-specific siRNA (sip63)-transfected primary keratinocytes. Downregulation of ΔNp63 by specific siRNA increased the levels of TSLPR and IL-7Rα mRNAs. Student's t test. *P<0.05 and ***P<0.005 versus control. Data are representative of three independent experiments. (TIFF) [file pone.0105498.s002.tiff]

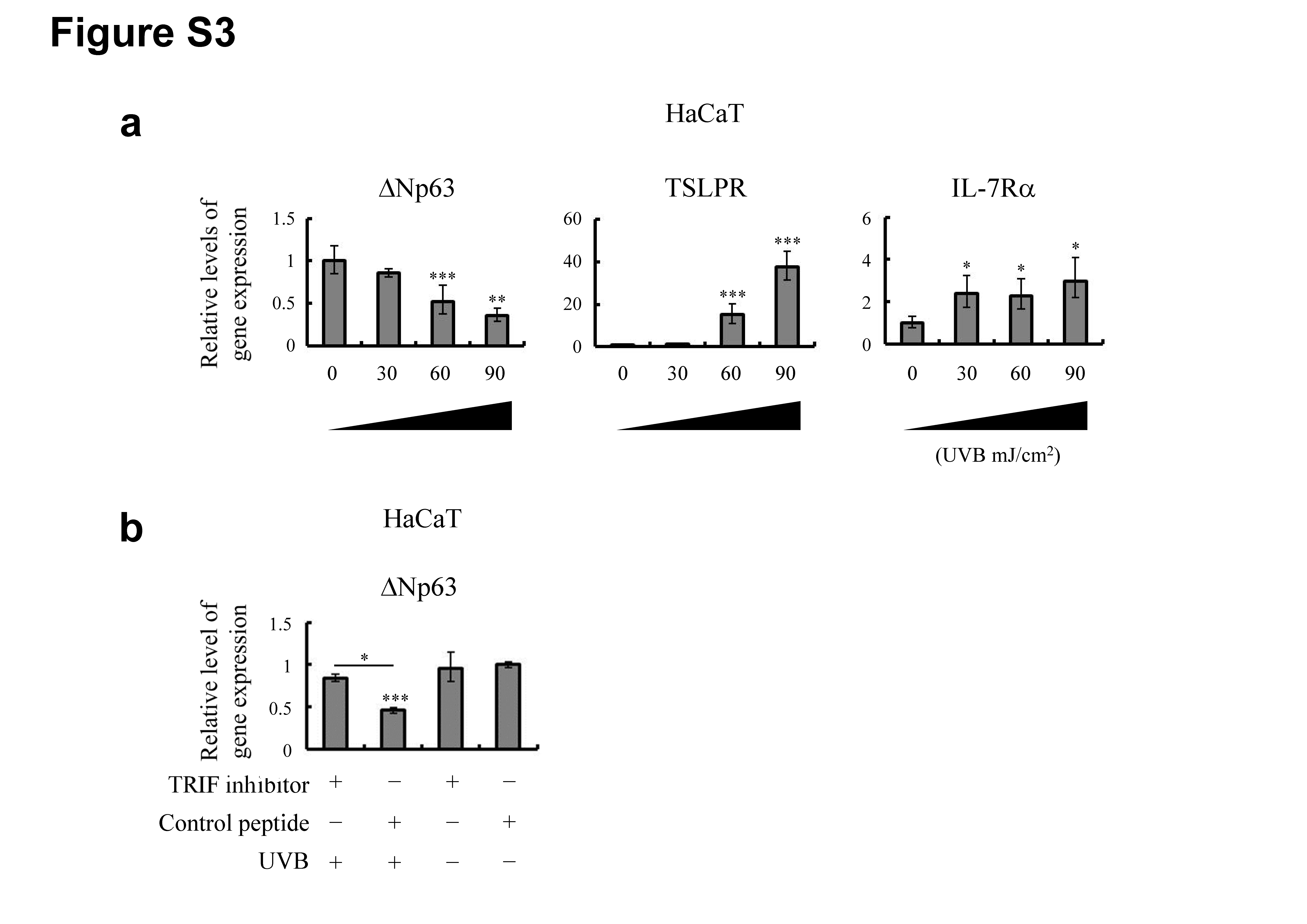

Supplement: Figure S3 — Effects of ultraviolet B (UVB) irradiation on HaCaT keratinocytes. (a) Quatitative RT-PCR showing the levels of ΔNp63, TSLPR and IL-7Rα mRNAs in UVB-exposed HaCaT keratinocytes. UVB exposure simply reduced the level of ΔNp63 and concomitantly upregulated the levels of TSLPR and IL-7Rα mRNAs. (b) Quantitative RT-PCR confirming whether inhibition of Toll/interleukin-1 receptor domain-containing adaptor protein inducing interferon β (TRIF) rescues downregulation of ΔNp63 mRNA by UVB exposure in HaCaT keratinocytes. TRIF inhibitor rescued ΔNp63 downregulation by UVB exposure. One-way ANOVA followed by Tukey's multiple-comparison test.*P<0.05, **P<0.01 and ***P<0.005 versus control. Data are representative of at least three independent experiments. (TIFF) [file pone.0105498.s003.tiff]

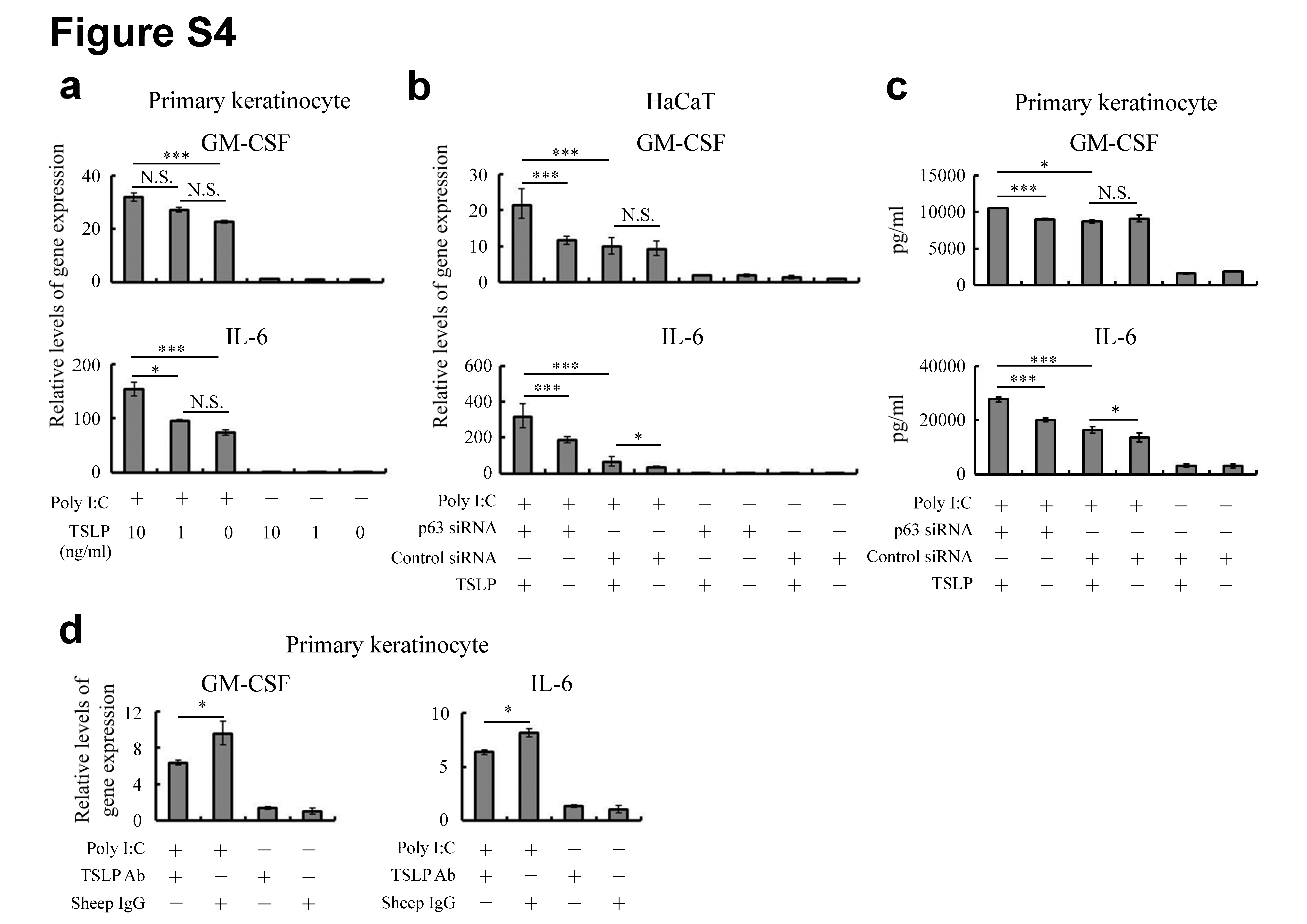

Supplement: Figure S4 — Expression of inflammatory cytokines in ΔNp63-deficient keratinocytes is enhanced by TSLP stimulation. (a) Quantitative RT-PCR demonstrating the levels of granulocyte colony-stimulating factor (GM-CSF) and IL-6 mRNAs at 24 hr after treatment with exogenous TSLP (1 or 10 ng/ml) under the condition of TLR3 stimulation (5 µg/ml poly I:C) in primary keratinocytes. Treatment with exogenous TSLP increased the expressions of these cytokines under the condition of TLR3 stimulation. (b) Quantitative RT-PCR showing the levels of GM-CSF and IL-6 mRNAs at 24 hr after stimulation with 10 µg/ml poly I:C and 10 ng/ml TSLP in siControl and sip63 HaCaT keratinocytes. Stimulation with TLR3 and TSLP enhanced the expressions of these cytokines in ΔNp63-deficient HaCaT keratinocytes. (c) ELISA demonstrating GM-CSF and IL-6 release at 48 hr after treatment with 5 µg/ml poly I:C and 10 ng/ml TSLP in culture supernatant from siControl and sip63 primary keratinocytes. Down regulation of ΔNp63 and/or exogenous TSLP increase GM-CSF and IL-6 release under the condition of TLR3 stimulation. (d) Quantitative RT-PCR confirming whether neutralization of TSLP inhibits the generation of GM-CSF and IL-6 under the condition of TLR3 stimulation (5 µg/ml poly I:C) in primary keratinocytes. Neutralization of TSLP inhibits the expressions of these cytokines under the condition of TLR3 stimulation. One-way ANOVA followed by Tukey's multiple-comparison test. *P<0.05, ***P<0.005 and N.S., not significant. Data are representative of at least three independent experiments. (TIFF) [file pone.0105498.s004.tiff]

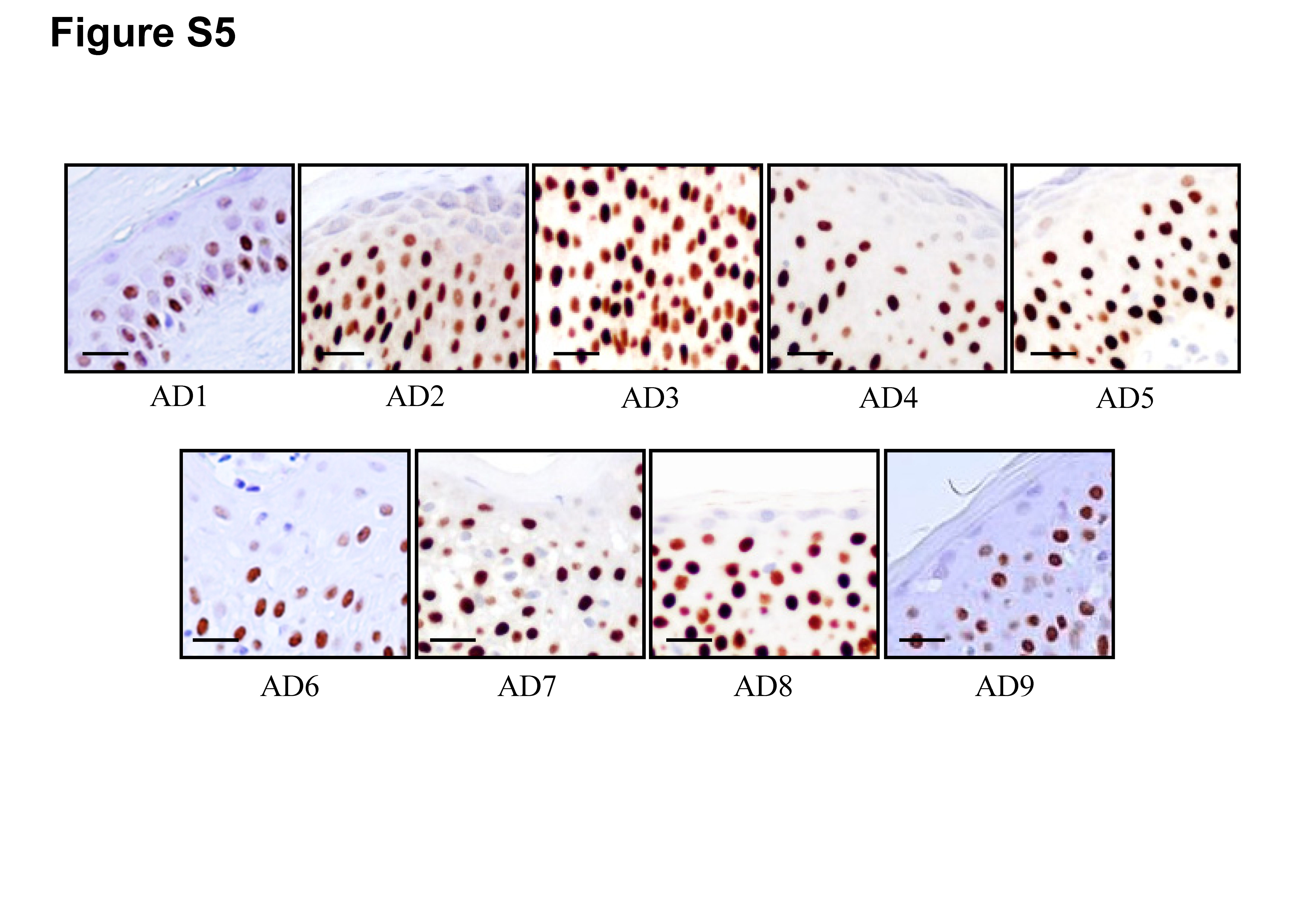

Supplement: Figure S5 — Representative figures of ΔNp63-immunostaining of AD skin lesions. Paraffin-embedded tissue sections of 10 cases of AD lesions (AD1 to AD10) were examined for the distribution of ΔNp63. AD10 is depicted in Figure 5a and the cases of AD1 to AD9 are shown here. Bar = 20 µm. (TIFF) [file pone.0105498.s005.tiff]

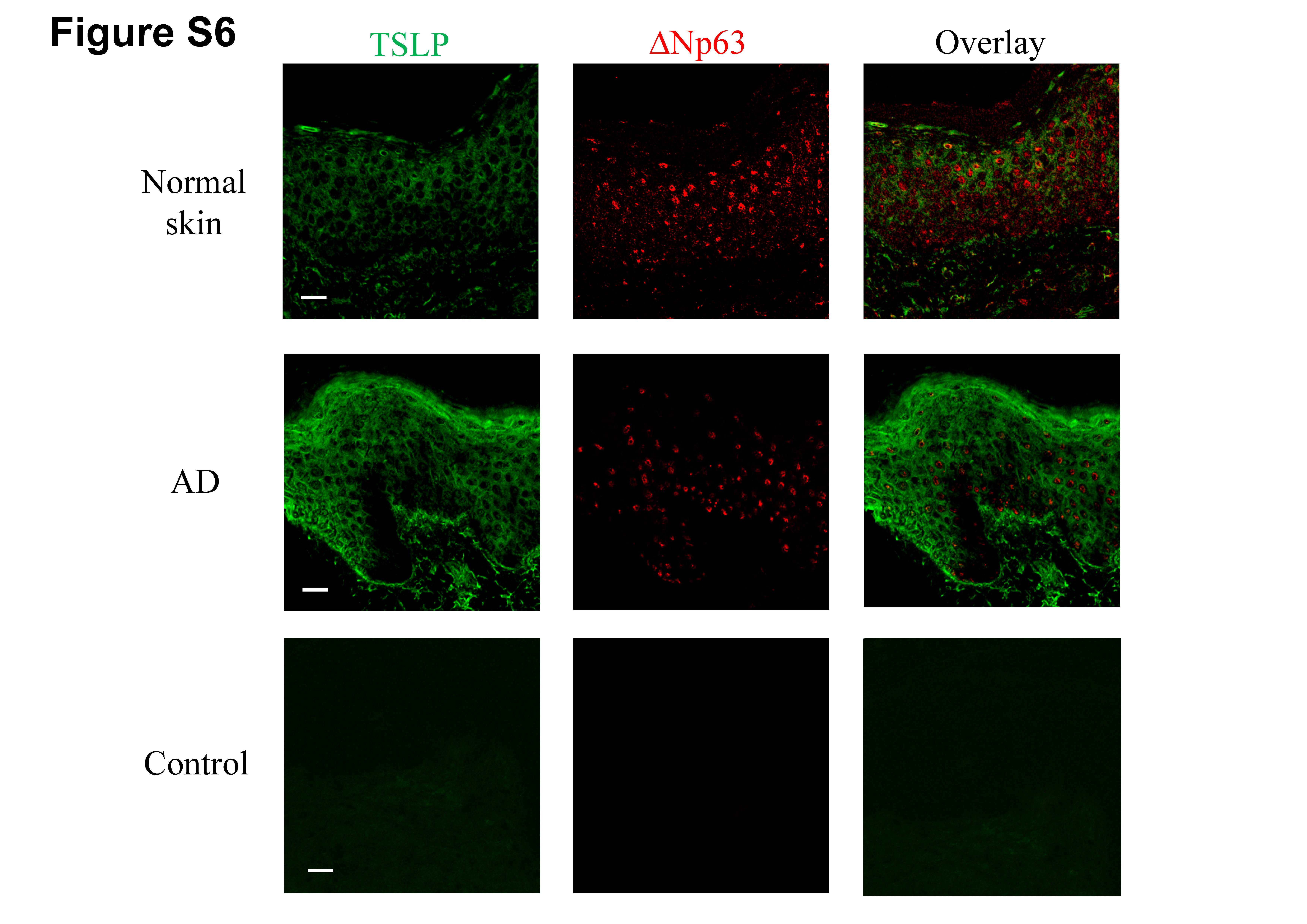

Supplement: Figure S6 — Immunofluorescence labeling of ΔNp63 and TSLP in AD and normal skin. TSLP was overexpressed in the skin from a patient with AD compared to the level in normal skin. On the other hand, keratinocytes in the skin from a patient with AD expressed a lower level of ΔNp63 than that in normal keratinocytes. Bar = 20 µm. (TIFF) [file pone.0105498.s006.tiff]
